# Les alertes de thrombolyse des accidents vasculaires cérébraux ischémiques: expérience de la clinique internationale Al Badie au secteur libéral de Fès (étude transversale de 60 cas)

**DOI:** 10.11604/pamj.2024.47.167.42376

**Published:** 2024-04-04

**Authors:** Imane Najmi, Saber Janati Idrissi, Khadija Bensouda, Zineb Marzouki, Mohammed Dinia, Ilyass Talbi, Soumaya Benmaamar, Siham Bouchal, Samira El Fakir, Karima El Rhazi, Oussama Fassi Fihri, Mohammed Faouzi Belahsen

**Affiliations:** 1 Unité Neurovasculaire, Clinique Internationale Al Badie, 355 Lotissement Jardins El Badie, Ain Chkf, 30000 Fès, Maroc,; 2 Service des Urgences, Clinique Internationale Al Badie, Fès, Maroc,; 3 Service de Radiologie, Clinique Internationale Al Badie, Fès, Maroc,; 4 Service de Cardiologie et de Cardiologie Interventionnelle, Clinique Internationale Al Badie, Fès, Maroc,; 5 Service de Réanimation, Clinique Internationale Al Badie, Fès, Maroc,; 6 Laboratoire d'Epidémiologie, Recherche Clinique et Science Communautaire, Faculté de Médecine et de Pharmacie, Université Sidi Mohamed Ben Abdellah, Fès, Maroc,; 7 Service de Neurologie, Centre Hospitalier Universitaire Hassan II Fès, Fès, Maroc

**Keywords:** Accident vasculaire cérébral ischémique, alerte de thrombolyse, thrombolyse intraveineuse, tenecteplase, Ischemic stroke, thrombolysis alert, intravenous thrombolysis, tenecteplase

## Abstract

La thrombolyse intraveineuse est le traitement de référence de l'accident vasculaire cérébral (AVC) ischémique aigu. Nous reportons dans cette étude le circuit des patients au cours de l´alerte de thrombolyse au secteur libéral au Maroc. C´est une étude prospective portant sur tous les patients, admis aux urgences de la clinique privée internationale Al Badie depuis janvier 2022 jusqu´en septembre 2023, pour un déficit neurologique d´installation brutale dans un délai de 12 heures. Les caractéristiques épidémiologiques, cliniques et étiologiques ainsi que les délais extra et intra-hospitaliers étaient recueillis. Soixante patients étaient inclus dans l´étude. Le délai d´admission moyen des patients était de 198,36 ± 79,23 minutes. Le score NIHSS (National Institutes of Health Stroke Scale) moyen était de 10,41 ± 4,97. Le délai moyen d´imagerie était de 26,68 ± 9,63 minutes. Le diagnostic d´AVC ischémique était le plus fréquent (85%) suivi des « stroke mimics » (11.6%). Treize patients ont bénéficié d´une thrombolyse par la tenecteplase. Le délai moyen entre l´admission et le début de la thrombolyse était de 107,15 ± 24,48 minutes. L´imagerie de contrôle à 24 heures de la thrombolyse a objectivé une transformation hémorragique symptomatique chez 3 patients. Six patients étaient transférés au CHU Hassan II pour thrombolyse et/ou thrombectomie mécanique. Après 3 mois, 4 patients étaient autonomes (score de Rankin modifié entre 0 et 2). Notre expérience montre qu´il s´avère impératif d´améliorer les délais extra et intra-hospitaliers de prise en charge afin d´augmenter la proportion des patients thrombolysés.

## Abstract (en)

Intravenous thrombolysis is the standard treatment for acute ischemic stroke. We here report the cases of thrombolysis alert in the private sector in Morocco We conducted a prospective study of all patients with neurological deficit of sudden onset occurred within the first 12 hours admitted to the Emergency Department of the Al Badie international private clinic from January 2022 to September 2023. Epidemiological, clinical and etiological characteristics as well as data on outpatient and inpatient delays were collected. Sixty patients were included in the study. The average admission delay was 198.36 ± 79.23 minutes. The mean NIHSS (National Institutes of Health Stroke Scale) score was 10.41 ± 4.97. The average time for imaging was 26.68 ± 9.63 minutes. Ischaemic stroke was the most common diagnosis (85%), followed by “stroke mimics” (11.6%). Thirteen patients underwent thrombolysis with tenecteplase. The mean time from admission to the initiation of thrombolysis was 107.15 ± 24.48 minutes. Follow-up imaging at 24 hours post thrombolysis revealed symptomatic haemorrhagic transformation in 3 patients. Six patients were transferred to the Hassan II University Hospital for thrombolysis and/or mechanical thrombectomy. After 3 months, 4 patients were autonomous (Rankin score changed between 0 and 2). Our experience shows that it is imperative to reduce outpatient and inpatient delays in treatment in order to increase the proportion of patients treated with thrombolysis.

## Introduction

L'accident vasculaire cérébral ischémique (AVCI) est une cause majeure d´handicap neurologique et représente un problème majeur de la santé publique. La reperfusion rapide par la thrombolyse intraveineuse (TIV) reste l´une des méthodes thérapeutiques de référence les plus efficaces [1]. L´incidence et la mortalité des accidents vasculaires cérébraux (AVC) auraient augmenté aux pays du Moyen-Orient et d´Afrique du Nord (MENA) au cours de la dernière décennie [2]. Au Maroc, l´incidence des AVC continue à augmenter au fil des années et le sous-type ischémique représente 85% de tous les AVC [3]. L´alerte de thrombolyse (AT) permet l´entrée du patient présentant des symptômes évoquant un AVC dans la filière de prise en charge optimale dans les meilleurs délais. A Fès, l´une des plus grandes villes du Maroc avec une population de 1.29 million habitants, la procédure de l´AT était disponible uniquement au niveau du centre hospitalier universitaire (CHU) Hassan II où elle était initiée en avril 2010 [4]. Depuis janvier 2022, une filière AT est instaurée dans une clinique privée au secteur libéral à Fès. L´objectif de notre étude est d´évaluer le circuit des patients se présentant pour suspicion d´AVC au cours de l´AT au secteur libéral avec les délais de prise en charge ainsi que de déterminer les principaux obstacles à optimiser et les ajustements à réaliser.

## Méthodes

**Cadre de l´étude**: notre étude est réalisée au secteur libéral de Fès portant sur 60 patients admis aux urgences d´une clinique privée à Fès (clinique internationale Al Badie). Les patients étaient prospectivement recrutés, à travers une base de données neurovasculaires, crée au sein de la clinique sur une période de 21 mois allant de janvier 2022 jusqu´à septembre 2023.

**Type de l´étude**: notre étude est transversale.

**Participants à l´étude**: tous les patients se présentant pour un déficit neurologique d´installation brutale dans un délai de 12 heures, chez qui une AT a été déclenchée, étaient inclus dans l´étude. Les patients admis pour un déficit neurologique, chez qui une AT n´a pas été déclenchée, étaient exclus de l´étude.

**Conception de l´étude**: depuis l´instauration de la filière des AT en janvier 2022, un circuit du patient au cours de l´AT au sein de la clinique a été établi et une unité neurovasculaire avec une équipe multidisciplinaire (urgentistes, neurologue, radiologue, cardiologues et réanimateurs) ont été développés au sein de la clinique pour la prise en charge des patients admis pour un AVC aigu. Une fois que le patient arrive aux urgences, il est initialement évalué par le médecin urgentiste qui confirme l´origine neurologique du déficit et l´heure du début des symptômes. Ainsi, il déclenche l´AT. Le neurologue est immédiatement avisé et une imagerie cérébrale est lancée après passage des procédures administratives. Le patient bénéficie d´une mise en condition (scope, prise de la tension artérielle et de la glycémie, la fréquence cardiaque, la fréquence respiratoire, la saturation en oxygène, la température, pose d´une voie veineuse périphérique). Un électrocardiogramme est réalisé. Un bilan biologique est aussi lancé en urgence incluant le dosage de l´INR (International Normalized Ratio) pour les patients sous anticoagulation orale par les antivitamies K (AVK). Le neurologue recueille les antécédents du patient et son histoire de la maladie. Puis, il évalue le déficit neurologique en utilisant le score NIHSS (National Institutes of Health Stroke Scale). Un déficit mineur est défini par un score NIHSS inférieur à 6.

Le patient est transféré immédiatement au service de radiologie où il va bénéficier d´une imagerie cérébrale par résonnance magnétique (IRM) comportant des séquences de diffusion (*Diffusion-weighted imaging*), FLAIR (*Fluid Attenuated Inversion Recovery*), SWI (*Susceptibility Weighted Imaging*) et TOF (*Time Of Flight*). Le neurologue et radiologue analysent toutes les séquences de l´IRM. Un infarctus cérébral qui apparait en hypersignal sur la séquence de diffusion sans traduction sur la séquence FLAIR défini un infarctus aigu dans le délai pour la thrombolyse. La localisation et la taille de l´infarctus, ainsi que le niveau d´occlusion et la taille du thrombus, sont aussi analysés. Le neurologue évalue les critères d´inclusion et d´exclusion de la thrombolyse intraveineuse. Si l´indication de la TIV est retenue, le patient ou sa famille fournissent leur consentement écrit avant l´administration du traitement thrombolytique, il passe les procédures administratives et il est ensuite transféré à l´unité de soins intensifs neurovasculaire. Les patients pour lesquels une AT a été déclenchée et chez qui l´IRM cérébrale n´a pas objectivé d´infarctus, étaient considérés comme des « stroke mimics ». Nous avons calculé le nombre des AT déclenchées ainsi que le nombre des TIV réalisées. Nous avons recueillis les données suivantes: l´âge, le sexe, le délai entre le début des symptômes et l´admission aux urgences, le score NIHSS initial, les résultats du bilan biologique, le délai entre l´admission et l´imagerie cérébrale, le type d´imagerie réalisée, le diagnostic final, la performance ou non de la TIV, les raisons de non TIV. Pour les patients thrombolysés, nous avons aussi recueillis le délai entre l´imagerie et la TIV, le délai entre l´admission et la TIV, le délai entre le début des symptômes et la TIV, le produit thrombolytique utilisé avec la dose administrée, le score NIHSS après la TIV, les résultats de l´imagerie de contrôle à 24 heure de la TIV, la présence ou non d´une transformation hémorragique selon la classification ECASS II, le score de Rankin modifié à 3 mois de la TIV. Pour les patients transférés au CHU Hassan II, nous avons précisé les raisons du transfert et la réalisation ou non d´un geste de revascularisation (TIV et/ou thrombectomie mécanique).

**Analyse des résultats**: les moyennes avec les écarts types ont été utilisés pour les variables quantitatives. Les pourcentages ont été mesurés pour les fréquences des variables qualitatives. Nous avons utilisé le test t de Student pour comparer les moyennes et la corrélation de Pearson pour étudier la corrélation entre les délais. Pour tous les tests statistiques, le seuil de signification statistique p < 0.05 a été choisi. Toutes les analyses statistiques étaient réalisées en utilisant IBM SPSS (Statistical Package for Social Sciences) version 26.

**Clairance éthique**: le comité d´éthique hospitalo-universitaire de Fès a approuvé l´étude. Tous les patients inclus ont fourni un consentement éclairé écrit.

## Résultats

Durant la période d´étude, 223 patients ont consulté aux urgences pour suspicion d´AVC, parmi lesquels 60 patients (26,9%) étaient admis dans un délai de 12 heures et chez qui une AT a été déclenchée. Deux cent seize patients avaient un diagnostic final d´AVCI. Le Tableau 1 résume les caractéristiques démographiques, cliniques et étiologiques des patients admis en AT. La moyenne d´âge des patients était de 71 ± 10,93 ans. Un peu plus de cinquante-un (51,6%) des patients étaient de sexe féminin (sex ratio: 0,93). Le délai d´admission moyen des patients (du début des symptômes à l´arrivée aux urgences) était de 198,36 ± 79,23 minutes. Chez 6 patients, il s´agissait d´un déficit constaté au réveil ou de délai inconnu. Un peu plus de cinquante-cinq pourcent (55,7%) des patients étaient admis durant la journée. Vingt-et-un pourcent (21,3%) des patients étaient admis durant le week-end. Le score NIHSS moyen était de 10,41 ± 4,97 dont 36 patients (60%) avaient un score supérieur ou égal à 12. Tous les patients ont bénéficié d´une imagerie cérébrale par IRM. Le délai moyen d´imagerie (de l´admission à la salle d´IRM) était de 26,68 ± 9,63 minutes.

**Tableau 1:** caractéristiques démographiques, cliniques et étiologiques des patients

	Variables	Valeur, Nombre(pourcentage)
**Sexe**	Homme	29 (48,3%)
	Femme	31 (51,6%)
	Sex-ratio	0,93
**Age (ans)**	Moyenne ± DS	71 ± 10,93
	Intervalle	[38-95]
**Délai Début-Admission (minutes)**	Moyenne ± DS	198,36 ± 79,23
	Intervalle	[60-420]
**NIHSS**	Moyenne ± DS	10,41 ± 4,97
	Intervalle	[2-20]
	<12	24 (40%)
	≥12	36(60%)
**Délai Admission-Imagerie (minutes)**	Moyenne ± DS	26,68 ± 9,63
	Intervalle	[10-50]
**Horaire**	Journée	34(56,6%)
	Nuit	26 (43,3%)
**Week-end**	Oui	12 (20%)
	Non	48 (80%)
**Etiologies**	AVC ischémique	51 (85%)
	AVC hémorragique	2 (3,3%)
	Stroke mimics	7 11,6%)
**Thrombolyse intraveineuse**	Oui	13(21,6%)
	Non	47(78,4%)

NIHSS: *National Institutes of Health Stroke Scale*; DS: dérivation standard; AVC: accident vasculaire cérébral

Le diagnostic d´AVCI était retenu chez 51 patients (85%), d´AVC hémorragique chez 2 patients (3,3%) et de « stroke mimics » chez 7 patients (11,6%). Les principales étiologies des « stroke mimics » dans notre série étaient les crises d´épilepsie (3 patients), les tumeurs cérébrales (2 patients), l´hémorragie cérébrale (2 patients), l´hypoglycémie (1 patient) et le trouble conversif (1 patient). Parmi les 60 AT, 13 patients ont bénéficié d´une TIV au sein de l´unité de soins intensifs neurovasculaires de la clinique, ce qui représente une proportion de 25,4% des AVCI admis dans le délai de 12 heures et 21,6% de toutes les AT. Six patients (11,7%) parmi les AT étaient transférés aux urgences et/ou à l´unité neurovasculaire du CHU Hassan II. Les raisons du transfert étaient la programmation de thrombectomie pour un patient et pour complément de prise en charge pour les cinq autres. Les 41 AT (68,3%) restantes avaient des contrindications à la TIV. La principale raison de non thrombolyse était la présence d´un AVCI mineur sans occlusion visible (14 patients). Le Tableau 2 représente les raisons de non thrombolyse chez patients non thrombolysés. Le Tableau 3 résume les caractéristiques démographiques, cliniques des 13 patients thrombolysés ainsi que leur score de Rankin modifié après 3 mois de l´AVC. La moyenne d´âge des patients thrombolysés était de 76,15 ± 9,25 ans. Le délai d´admission moyen était de 186,15 ± 86,89 minutes. Le score NIHSS moyen avant la TIV était de 14,76 ± 4,17. Le délai moyen d´imagerie était de 29,07 ± 7,95 minutes. Le délai moyen de l´imagerie à la TIV était de 78.07 ± 20,87 minutes. Tous les patients étaient thrombolysés par la tenecteplase (dose moyenne 3,76 ± 3,72 ml). Le délai moyen de l´admission aux urgences au début de la TIV était de 107.15 ± 24.48 minutes. Le délai moyen du début des symptômes à la TIV était de 271,76 ± 99,88 minutes. Après la TIV, le score NIHSS moyen était de 10,41 ± 4,97. L´imagerie de contrôle après 24 heures de la TIV a objectivé une transformation hémorragique chez 5 patients (38,4%). La transformation hémorragique était symptomatique chez 3 patients. Après 3 mois, 4 patients étaient autonomes avec un Rankin < 2, cinq (5) patients avaient un Rankin entre 2 et 5. Quatre patients sont décédés dans les 7 jours suivant l´AVC dont la cause était la transformation hémorragique massive chez 2 patients et la décompensation d´une insuffisance cardiaque chez les 2 autres patients. Pour les 6 patients transférés au CHU Hassan II, un patient a bénéficié d´une TIV combinée à une thrombectomie mécanique, deux patients avaient bénéficié d´une TIV seule. Les 3 autres patients sont arrivés hors délai de la TIV devant la constatation d´un AVCI constitué après la réalisation d´un scanner cérébral récent aux urgences.

**Tableau 2:** raisons de non thrombolyse pour les patients présentant un AVC ischémique

Raisons de non thrombolyse	Nombre de patients (32)
AVC ischémique mineur sans occlusion	14
Occlusion totale de la carotide homolatérale	7
AVC ischémique constitué	6
Prise d´anticoagulants	3
AIT sans occlusion	2

AVC: accident vasculaire cérébral; AIT: accident ischémique transitoire

**Tableau 3:** caractéristiques épidémiologiques et cliniques des patients thrombolysés avec le Rankin à 3 mois

	Variables	Valeur, Nombre (pourcentage)
Age (ans)	Moyenne± DSIntervalle	76,15 ± 9,25 [53-88]
Délai Début-Admission (minutes)	Moyenne ± DS Intervalle	186,15 ± 86,89 [60-390]
NIHSS avant la TIV	Moyenne ± DS Intervalle	14,76 ± 4,17 [5-20]
D±lai admission-imagerie (minutes)	Moyenne ± DS Intervalle	29,07 ± 7,95 [15-45]
Délai imagerie-TIV (minutes)	Moyenne ± DSIntervalle	78.07 ± 20,87 [45-120]
Délai admission-TIV (minutes)	Moyenne ± DS Intervalle	107,15 ±24.48 [60-150]
Délai D±but-TIV (minutes)	Moyenne ± DS Intervalle	271,76 ± 99,88 [105-490]
NIHSS après la TIV	Moyenne ± DS Intervalle	10,41± 4,97[0-20]
Rankin à 3 mois	Intervalle	
	0-1	4(30,7%)
	2-5	5(38,4%)
	6	4 (30,7%)

DS: dérivation standard; NIHSS: *National Institutes of Health Stroke Scale*; TIV: thrombolyse intraveineuse

**Résultats analytiques**: nous n´avons pas trouvé une différence significative du délai d´admission aux urgences et du délai de la réalisation de l´imagerie entre les patients admis durant le week-end et ceux admis au cours de la semaine (222,30 ± 95,66 versus 191,87 ± 74.00, p=0,2 et 28,69 ± 9,51 vs 26,14 ± 9,69, p=0.4 respectivement). Nous n´avons pas trouvé une différence significative du délai d´admission aux urgences et du délai de la réalisation de l´imagerie entre les patients admis durant la journée et ceux admis durant la nuit (200,14 ± 86,25 vs 196,11 ± 70,95, p=0,8 et 27,91 ± 8,90 vs 25,14 ± 10,44, p=0.2 respectivement). Il n´y avait pas de différence significative entre la sévérité du déficit (NIHSS ≥12) et le délai de la réalisation de l´imagerie (25.38 ± 9.64 vs 28.56 ± 9,49, p=0,2). Il n´y avait pas de corrélation significative entre le délai d´admission et le délai de l´imagerie (r= 0,1, p=0,4). Il n´y avait pas de corrélation significative entre le délai d´admission et la TIV (r= -0,08, p=0,7). Il n´y avait pas de différence significative du délai de la TIV chez les patients admis durant le week-end et ceux admis au cours la semaine (p=0,7).

## Discussion

Notre étude est la première à évaluer le circuit du patient dans la filière des alertes de thrombolyse des AVCI aigus dans le secteur libéral au Maroc et en particulier dans la région de Fès. La proportion des TIV dans notre série était de 21,6% de toutes les AT et de 2,4% des AVCI admis dans les délais. Il faut environ 5 AT pour réaliser une TIV. Dans la série de Daouda *et al*. ayant exploré les AT du CHU Hassan II de Fès, 313 AT étaient incluses dans l´étude dont la proportion des TIV était de 14,7% des AT et de 20,9% des AVCI admis dans le délai. Il leur a fallu 6 à 7 AT pour réaliser une TIV [5]. Dans notre étude, nous avons constaté que le délai d´admission moyen était de 197,16 ± 78,68 minutes ainsi que le délai entre le début des symptômes et la TIV moyen était de 271,76 ± 95,96 minutes. Nos délais sont plus longs que ceux rapportés dans des études précédentes, allant de 51 à 90 minutes et de 128 à 155 minutes respectivement [6,7]. Daouda *et al*. avaient rapporté, dans la série AT réalisée au CHU de Fès, un délai d´admission moyen de 125,59 ± 62,78 minutes et un délai entre le début des symptômes et la TIV moyen de 184,39 ± 51,7 minutes [5]. Une analyse des résultats du registre SITS (Safe Implementation of Treatments in Stroke) dans la région du moyen orient et d´Afrique du nord (MENA) avait rapporté un délai d´admission médiane de 5 heures [2]. Le délai préhospitalier retardé reste la principale raison de non thrombolyse au Maroc ainsi que dans les pays en voie de développement. En effet, le délai préhospitalier moyen pour l´ensemble des patients admis pour un AVCI au Maroc varie entre 26 et 61,9 heures, ce qui dépasse largement la fenêtre thérapeutique recommandée par les essais cliniques randomisés [8,9]. Cette constatation est conforme au délai médian d'admission mentionné dans une revue de la littérature sur le continent africain qui est de 31 heures [10]. De plus, il rejoint le délai moyen de consultation rapporté dans une étude menée au Centre Hospitalier Sahloul de Sousse en Tunisie, où l'on faisait état d'un délai de 16 heures [11]. Ceci pourrait s´expliquer par le fait que la plupart des patients arrivaient aux urgences avec des moyens de transport personnels ou communs (taxi, voiture personnelle) et que seule une minorité utilisait les services d´ambulance (3,5%) [5,8]. Une particularité que nous avons constaté au secteur privé, c´est que les patients consultent d´abord leurs médecins traitants avant qu´ils soient référés aux urgences pour prise en charge. Aussi, le fait que seulement 26,9% des AVC consultent dans les délais pourrait être dû au fait que les patients ne savent pas que cette thérapeutique a été récemment implantée dans la clinique puisque la TIV était, pour longtemps, limitée à quelques CHU du Maroc en particulier le CHU de Fès.

La proportion des patients thrombolysés représente 6,01% de tous les AVCI de notre série. Daouda *et al*. ont rapporté une proportion de 2,8% de patients thrombolysés pour un AVCI aigu [5]. Aussi, Acherqui *et al*. ayant exploré l´éligibilité à la TIV des AVCI aigus dans le CHU de Casablanca, avait reporté un taux de TIV de 8,4% [12]. Toutes ces proportions de TIV rapportées dans les différents centres marocains sont faibles par rapport à celles rapportées dans des études précédentes allant de 15% dans la série de McCormick *et al*. à 16,7% dans la série de Schmidt *et al*. [13,14]. Par ailleurs, ce retard de consultation dans la population marocaine pourrait être amélioré par des campagnes de sensibilisation de la population et des praticiens [8,15,16]. Ce constat a été confirmé par une étude menée au CHU Mohamed VI de Marrakech, dans laquelle 59,8 % des personnes interrogées n'ont pu citer au moins un signe révélateur d'AVC [17]. Dans la même perspective, une étude récente a démontré que la faible sensibilisation aux signes et symptômes de l'AVC, le manque de transports médicaux, de personnel soignant et d'unités neurovasculaires, ainsi que le coût économique de l'accès à l'imagerie cérébrale et à la TIV étaient des obstacles majeurs à l´amélioration des soins et de la gestion de l´AVC en Afrique [10].

Le délai moyen d´imagerie était de 26,55 minutes. Notre délai est satisfaisant en comparaison avec les centres marocains qui ont rapporté un délai d´imagerie moyenne de 28 minutes au CHU de Fès et de 138 minutes au CHU de Casablanca [5,12]. Aussi, notre délai est proche du délai de 25 minutes rapporté par Sauser *et al*. [18]. Ceci pourrait s´expliquer par la sensibilisation continue du personnel de la clinique et la mobilisation rapide des médecins urgentistes et de l´équipe de radiologie pour faciliter la réalisation de l´imagerie. Cependant, il semble que la procédure administrative prolonge le délai de réalisation de l´imagerie dans notre structure. Nos patients ont bénéficié d´une IRM cérébrale qui est plus sensible et spécifique au cours des AT par rapport au scanner, ce qui permet une meilleure sélection des patients éligible à la TIV. Le délai moyen entre de l´admission aux urgences et le début de la TIV était de 98.69 minutes, proche de celui de CHU de Fès qui est de 90 minutes. Notre délai dépasse le délai recommandé qui est de 60 minutes [19]. Ceci est principalement lié aux procédures administratives. Le consentement du patient et/ou de sa famille était toujours rapidement obtenu après la réalisation de l´imagerie et la prise de décision de la TIV. Des assouplissements sont constamment instaurés à la clinique pour faciliter l´accès des patients à la TIV dans les plus brefs délais. Le score NIHSS initial moyen de nos patients avec un AVCI thrombolysé était de 14,7. Notre score est similaire à celui de Daouda *et al*. au CHU de Fès [5]. Cette similitude est évidente puisque nous recevons des patients de la même région. Cependant, nos patients sont plus graves par rapport aux séries du registre SITS-ISTR (*Safe Implementation of Treatments in Stroke-International Stroke Thrombolysis Register*) [20].

Nous avons constaté dans notre série les mêmes raisons de non thrombolyse rapportées par Bouchal *et al*. au service de neurologie du CHU de Fès [21]. Nous avons transféré 6 patients au CHU de Fès soit aux urgences soit directement au service de neurologie dont 3 patients ont bénéficié d´une TIV. Ceci reflète le modèle de collaboration entre les secteurs public et privé afin d´améliorer la qualité de prise en charge des patients. Nos patients étaient thrombolysés par la tenecteplase. Il est prouvé actuellement que l'utilisation de la tenecteplase est cliniquement justifiée et globalement similaire en termes d'efficacité et de sécurité à celle de l´Alteplase dans la prise en charge des AVCI aigus. La facilité d'administration (un simple bolus intraveineux et aucune perfusion d'entretien) pourrait offrir un avantage à la tenecteplase par rapport à l'Alteplase dans la population marocaine présentant des délais retardés de prise en charge [22-24]. Notre étude présente une principale limitation. Le recueil des délais extra-hospitaliers était fait par l´interrogatoire des patients ou leurs familles, ce qui peut aboutir à certaines incertitudes des délais.

## Conclusion

Nous pensons que le développement des unités neurovasculaires s´avère impératif pour améliorer la prise en charge des patients se présentant pour un AVCI aigu. Le secteur libéral pourrait également contribuer à l´amélioration et l´optimisation de cette prise en charge. Aussi, des formations en neurovasculaire, notamment à travers des diplômes universitaires, pourrait nettement augmenter le nombre des spécialistes neurovasculaires au Maroc et en particulier au secteur libéral. Avec la généralisation de l´assurance maladie obligatoire au Maroc, les actes de revascularisation (thrombolyse et thrombectomie) devraient être remboursés, ce qui permettrait également d´améliorer l´accès à ces thérapeutiques.

### Etat des connaissances sur le sujet

- *Taux de thrombolyse des AVC ischémiques au Maroc est très faible;*
- *Le délai d´admission est la principale raison de non thrombolyse au Maroc;*
- *La tenecteplase a prouvé son efficacité dans la thrombolyse des AVC ischémiques*.

### Contribution de notre étude à la connaissance

- *Première étude évaluant la filière des alertes de thrombolyse au secteur libéral au Maroc;*
- *L´IRM permet de proposer des thrombolyses au-delà de 4,5 heures car elle permet une meilleure analyse du parenchyme cérébral;*
- *Identification des principaux obstacles à optimiser et les ajustements à réaliser pour augmenter le taux de thrombolyse au secteur libéral du Maroc*.

## Remerciements

Nous souhaitons remercier les personnels du service de radiologie, ainsi que toute l´équipe paramédicale de la clinique pour leur coopération au cours de cette étude.
